# Silica Modified by Alcohol Polyoxyethylene Ether and Silane Coupling Agent Together to Achieve High Performance Rubber Composites Using the Latex Compounding Method

**DOI:** 10.3390/polym10010001

**Published:** 2017-12-21

**Authors:** Junchi Zheng, Xin Ye, Dongli Han, Suhe Zhao, Xiaohui Wu, Youping Wu, Dong Dong, Yiqing Wang, Liqun Zhang

**Affiliations:** 1 Engineering Research Center of Elastomer Materials on Energy Conservation and Resources, Ministry of Education, Beijing 100029, China; 2015400076@mail.buct.edu.cn (J.Z.); yexin@mail.buct.edu.cn (X.Y.); handl@mail.buct.edu.cn (D.H.); zhaosh@mail.buct.edu.cn (S.Z.); wuxiaohui555@163.com (X.W.); wuyp@mail.buct.edu.cn (Y.W.); 2 State Key Laboratory of Organic-Inorganic Composites, Beijing University of Chemical Technology, P.O. Box 57, Beisanhuan East Road, Beijing 100029, China; 3 Red Avenue New Materials Group Co., Ltd., Shanghai 200120, China; dondan68@163.com

**Keywords:** chemical and physical interface, surface modification of silica, latex compounding method, silica/NR composite

## Abstract

The study of preparing silica/rubber composites used in tires with low rolling resistance in an energy-saving method is fast-growing. In this study, a novel strategy is proposed, in which silica was modified by combing alcohol polyoxyethylene ether (AEO) and 3-mercaptopropyltriethoxysilane (K-MEPTS) for preparing silica/natural rubber (NR) master batches. A thermal gravimetric analyzer and Raman spectroscopy results indicated that both AEO and K-MEPTS could be grafted on to the silica surface, and AEO has a chance to shield the mercaptopropyl group on K-MEPTS. Silica modified by AEO and K-MEPTS together was completely co-coagulated with the rubber in preparing silica/NR composites using the latex compounding method with the help of the interaction between AEO and K-MEPTS. The performance of composites prepared by silica/NR master batches was investigated by a rubber process analyzer (RPA), transmission electron microscopy (TEM) and a tensile tester. These results demonstrate that AEO forms a physical interface between silica and rubber, resulting in good silica dispersion in the matrix. K-MEPTS forms a chemical interface between silica and rubber, enhancing the reinforcing effect of silica and reducing the mutual friction between silica particles. In summary, using a proper combination of AEO and K-MEPTS is a user-friendly approach for preparing silica/NR composites with excellent performance.

## 1. Introduction

Silica is a non-carbon filler that is an extremely important reinforcing filler in the rubber industry [1]. Previous studies have confirmed that silica combines good mechanical performance [2], high wet grip resistance [3] and low rolling resistance [4] for silica/rubber composites. At present, silica/rubber composites are commonly used for producing “green tires” [5,6], which have low rolling resistance, resulting in reducing the vehicle’s fuel consumption.

As an inorganic particle, silica has many hydroxyl groups (–OH) on its surface [7], causing the hydrophilic nature of the silica particle [8,9,10]. Therefore, the silica particle is less compatible with a hydrophobic polymer, such as rubber [11]. However, the silica surface can be modified due to the numerous reactive hydroxyl groups [12,13]. Silica modification is an effective method for improving the compatibility between silica and rubber. Treatment with reactive silane coupling agent (SCA) is one of the major methods used in silica modification [14,15]. In principle, SCA possesses a readily hydrolyzable alkoxy group that reacts with the hydroxyl groups on the silica surface to form a stable siloxane linkage [16]. In addition, some surface active agents (SAA), such as poly ethylene glycol, triethanolamine and cetyltrimethylammonium bromide, are commonly used in silica modification. SAA can absorb on the silica surface, resulting in covering the silica surface and reducing the amount of the exposed hydroxyl groups.

Natural rubber (NR), which contains 93%–95% *cis*-1,4-polyisoprene, is an essential biosynthesized polymer [17]. It is naturally found in the form of a colloidal system known as NR latex, in which rubber particles are dispersed in an aqueous medium [18,19]. Therefore, the latex compounding method is used to prepare silica/NR composites to address the problems of low efficiency in the outdated mechanical blending method [20,21].

Preparing a silica/NR slurry with good silica dispersion and stable NR particles is key for the latex compounding method mentioned above. The silica in this slurry should be completely co-coagulated with the rubber when preparing silica/NR master batches, which is a floc containing silica and rubber. As these materials work at a high frequency of motion, silica/rubber composites should have excellent dynamic performance, which is not required in most kinds of polymer composites. Therefore, the silica in the master batches should have the potential to form a chemical interaction with NR, because the internal friction of silica/NR composites, which is the major factor in dynamic performance such as rolling resistance and wet grip of tires, can be reduced by a chemical interaction between silica and rubber. Preparing modified silica, using a proper strategy to achieve an ideal organic silica surface, is the most practical process to meet the above requirements.

In previous research, sulfide-containing SCAs were used for silica modification when preparing silica/rubber composites using the latex compounding method [21,22,23,24]. In principle, sulfide-containing SCAs react with silica as mentioned above, resulting in improving the dispersion of silica in a latex system and rubber matrices. Meanwhile, sulfide-containing SCAs can react with the double bonds in rubber molecules by their sulfide group. Therefore, the structure by which rubber molecules and silica particles are linked by the SCA is formed between silica and rubber by the help of the sulfide-containing SCAs through a “coupling bridge” [25]. Previous researchers indicated that the “coupling bridge”, which is a typical chemical interface between silica and rubber, benefits the mechanical and dynamic properties of silica/rubber composites [26]. However, the hydroxyl groups on the silica react with the hydroxyl groups of hydrolyzed SCA in silica modified by SCA in the aqueous phase [27], meaning the hydrolysis of SCA is a precursor for silica modification. Polycondensation also occurs among the hydroxyl groups of hydrolyzed SCA, producing the polycondensates of SCA, resulting in the aggregation of several SCA molecules [25]. Therefore, using only SCA in silica modification is not adequately efficient. Only the chemical interface existing between silica and rubber is detrimental to the stretching of the rubber molecular chain under external force [28,29,30]. Moreover, the reaction between rubber and sulfide-containing SCA, known as “scorchy” behavior, is inevitable during the process [31], even though the mixing time and temperature are precisely controlled. Therefore, using sulfide-containing SCA only for silica modification is not an ideal method for preparing silica/rubber composites.

Parts of SAAs directly modify silica in the aqueous phase without polycondensation, and not all SAAs react with rubber under all conditions. Therefore, a physical interface between silica and rubber can only be formed with the help of SAA [32,33,34]. However, the dynamic performance of silica/rubber composites is very poor in the absence of a chemical interface between silica and rubber [8,28]. In our previous research, silica modified by SAA was not completely co-coagulated with the rubber when preparing silica rubber master batches using the latex compounding method.

For rubber composites prepared for “green tires”, the dynamic performance is extremely important. Therefore, for this research, a chemical interface between silica and rubber was formed. Preparing high performance silica/rubber composites for “green tires” using the latex compounding method is a more complex and restrictive study than the preparation of other polymer composites. Using different modifiers to form chemical and physical interfaces between silica and rubber is a simple and feasible method to achieve this goal.

**Figure 1:**
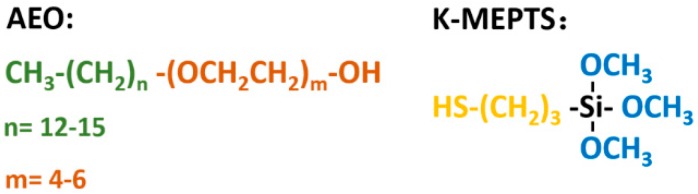
Chemical structure of alcohol polyoxyethylene ether (AEO) and 3-mercaptopropyltriethoxysilane (K-MEPTS).

In this research, the silica/NR master batches were prepared using the latex compounding method. Alcohol polyoxyethylene ether (AEO) [35]), a widely-used nonionic SAA, and 3-mercaptopropyltriethoxysilane (K-MEPTS) [36], which was commercially developed and widely used in the rubber industry, were selected as modifiers to be used together in silica modification in the aqueous phase (as shown in Figure 1). This is a novel strategy proposed for silica modification for preparing silica/rubber master batches. The magnitude of the chemical and physical interface between silica and rubber is varied by adjusting the amount of AEO and K-MEPTS used in silica modification. In our research, pure and different modified silica were characterized by Fourier transform infrared spectroscopy (FT-IR), thermal gravimetric analysis (TGA), and Raman spectroscopy. The interaction between K-MEPTS and AEO was confirmed through the results of these characterizations. Pure and different modified silica were used in preparing silica/NR master batches by the latex compounding method. In this part of the research, the role played by K-MEPTS and AEO using the co-coagulation of silica and NR was confirmed. Finally, the properties of silica/NR composites containing different modified silica were compared. The effect of the chemical interface between silica and rubber, formed by K-MEPTS, and the physical interface between silica and rubber, formed by AEO, on the performance of silica/NR composites was investigated.

## 2. Experimental Materials and Methods

### 2.1. Materials

High-ammonia NR latex with 60% total solid content was purchased from Hainan Rubber Industry Group Co., Ltd. (Hainan, China). Precipitated silica water slurry of K-160 (nanoparticle size: 20–30 nm, Brunauer-Emmett-Teller (BET) specific surface: 160.06 m^2^/g) was produced by Wilmar China (Jiamusi, China). AEO (average molecular weight: 421 g/mol), which possesses a terminal hydroxyl group that can react with hydroxyl groups on the silica surface and possesses a long molecular chain that consists of polyolefin and polyether, was prepared by BASF SE. K-MEPTS (molecular weight: 196 g/mol) was obtained from Nanjing Capatue Chemical Co., Ltd. (Nanjing, China). The rest of the required materials were commercially available.

### 2.2. Preparation of Modified Silica

The silica slurry solid content was measured to dilute to a 10% concentration (e.g., 100 g dry weight of silica for every 1000 g silica slurry) by adding water into the raw precipitated silica slurry. The silica water slurry was subjected to high-speed stirring (800 rpm) for 30 min to obtain a stable suspension. Five beakers, numbered 1–5, were prepared, and 1000 g of silica slurry were transferred to each beaker. All silica slurries were heated to a temperature of 70 °C under high-speed stirring (800 rpm). K-MEPTS (6, 6, 4, and 2 g) was added into Beakers 1–4, and AEO (4, 6, 8 and 8 g) was added into Beakers 2–5, respectively. The slurry was stirred for 0.5 h, and the modified silica slurry was then obtained. According to the amount of AEO and K-MEPTS added in the silica modification, we labelled the modified silica in Beakers 1–5 as A0K6-MS, A4K6-MS, A6K4-MS, A8K2-MS and A8K0-MS, respectively. The amounts of K-MEPTS and AEO used in the different modified silica are listed in Table 1.

Part of the pure silica and modified silica powder was obtained by drying the corresponding modified silica slurry. Pure silica and modified silica powders were extracted in a Soxhlet extractor using ethanol for 24 h, 15 min for each reflux, to remove un-grafted AEO and K-MEPTS. Then, all extracted silica powders were dried in the same oven at 70 °C for 24 h. These silica powders were prepared for characterization using FT-IR, TGA and Raman spectroscopy.

### 2.3. Preparation of Master Batches

The modified silica slurry was cooled to room temperature and blended with the NR latex. The solid content of the NR latex was confirmed in advance, and the weight ratio of silica to NR was 50:100 (e.g., 50 g of silica nanoparticles for every 100 g solid content of NR). Then, the mixture of silica and NR latex was stirred for 0.5 h and coagulated with 3% formic acid solution. Finally, the flocs were washed with water 6 times and then dehydrated in a drying oven at 60 °C for 36 h to obtain silica/NR master batches. The master batches prepared with A0K6-MS, A4K6-MS, A6K4-MS, A8K2-MS and A8K0-MS were called A0K6-MB, A4K6-MB, A6K4-MB, A8K2-MB and A8K0-MB, respectively.

### 2.4. Preparation of Silica/NR Composites

The formulation of silica/NR compounds is shown in Table 2. Silica/NR compounds were obtained through three stages of mixing. First, the master batches were masticated for 2 min in an internal mixer equipped with an oil circulating system to maintain the processing temperature at 55 °C. Then, zinc oxide, stearic acid and *N*-1,3-dimethylbutyl-*N*′-phenyl-*p*-phenylenediamine were added to the master batches successively. Second, compounds were kneaded for 5 min in the same internal mixer at 150 °C to further promote the reaction between silica and modifier and then naturally cooled to room temperature. Finally, *N*-cyclohexyl-2-benzothiazole-sulfenamide, diphenyl guanidine and sulfur were uniformly blended in sequence with the cooled compound in a 6-inch mill (Shanghai Rubber Machinery Works No. 1, Shanghai, China) at room temperature. The total mixing time was no more than 15 min. The silica/NR compounds that contained A4K6-MB, A6K4-MB and A8K2-MB were denoted as A4K6-C, A6K4-C and A8K2-C, respectively.

The scorch time (*T*_10_) and optimum cure time (*T*_90_) of the compound were measured using a disc vulcameter. The compounds were vulcanized at 143 °C according to their optimum cure time (*T*_90_) in a standard mold to produce the silica/NR vulcanizates, which were stored at room temperature for at least 24 h before determining the performance.

### 2.5. Characterizations

The groups of pure and modified silica were characterized by Fourier transform infrared spectroscopy (FT-IR; Bruker Optik GmbH Co., Tensor 27, Ettlingen, Germany), using the absorption mode under a wave ranging from 4000–400 cm^−1^ with a resolution of 4 cm^−1^. The samples were pressed into pellets together with potassium bromide.

Raman spectra of pure and modified silica samples were recorded from 3200–2500 cm^−1^ on an inVia confocal Raman spectrometer (Renishaw PLC, Gloucestershire, UK) using a 514-nm laser beam. The power of a 514-nm argon ion excitation laser at the source is approximately 50 mW (highest power) and 20 mW at the surface of the sample. The Raman spectra of the samples were obtained from pressed solid samples in a sealed capillary tube.

Weight loss measurements of pure and modified silica and the master batches were performed on a thermal gravimetric analyzer (TGA) STARe system (Mettler-Toledo Co., Greifensee, Switzerland) in a nitrogen atmosphere. Samples for the TGA tests were heated at a heating rate of 10 °C/min. The residual weight of master batches, NR, silica, K-MEPTS and AEO were recorded as *R*_m_, *R*_r_, *R*_s_, *R*_5_ and *R*_A_, respectively.

The filler dispersion in silica/NR master batches and silica/NR composites was observed under a Tecnai G2 20 transmission electron microscope (TEM, FEI Co., Hillsboro, OR, USA) with an accelerating voltage of 200 kV. Thin sections for TEM observations were cut by a microtome at −100 °C and collected on copper grids.

The dynamic rheological performances of silica/NR compounds and silica/NR composites were analyzed using RPA2000 (Alpha Technologies Co., Ltd., Akron, OH, USA) at 60 °C. For the rubber compounds, the strain varied from 0.1%–400% at the test frequency of 1 Hz. For the rubber vulcanizates, the strain varied from 0.1%–40% at the test frequency of 1 Hz. The test of each specimen was repeated 3 times.

The vulcanization characteristics of silica/NR compounds were measured at 143 °C using a P3555B2 disc vulkameter (Beijing Huanfeng Chemical Machinery Trial Plant, Beijing, China). The test of each specimen was repeated 3 times.

The mechanical performance of the silica/NR composites were investigated according to ASTM D638 specifications using a CMT4104 electrical tensile tester (Shenzhen SANS Test Machine Co., Shenzhen, China) with an across-head speed of 500 mm/min. The test of each specimen was repeated 5 times.

## 3. Results and Discussion

### 3.1. Characterization of Silica Modified by AEO and K-MEPTS

#### 3.1.1. FT-IR of Pure and Modified Silica

**Figure 2:**
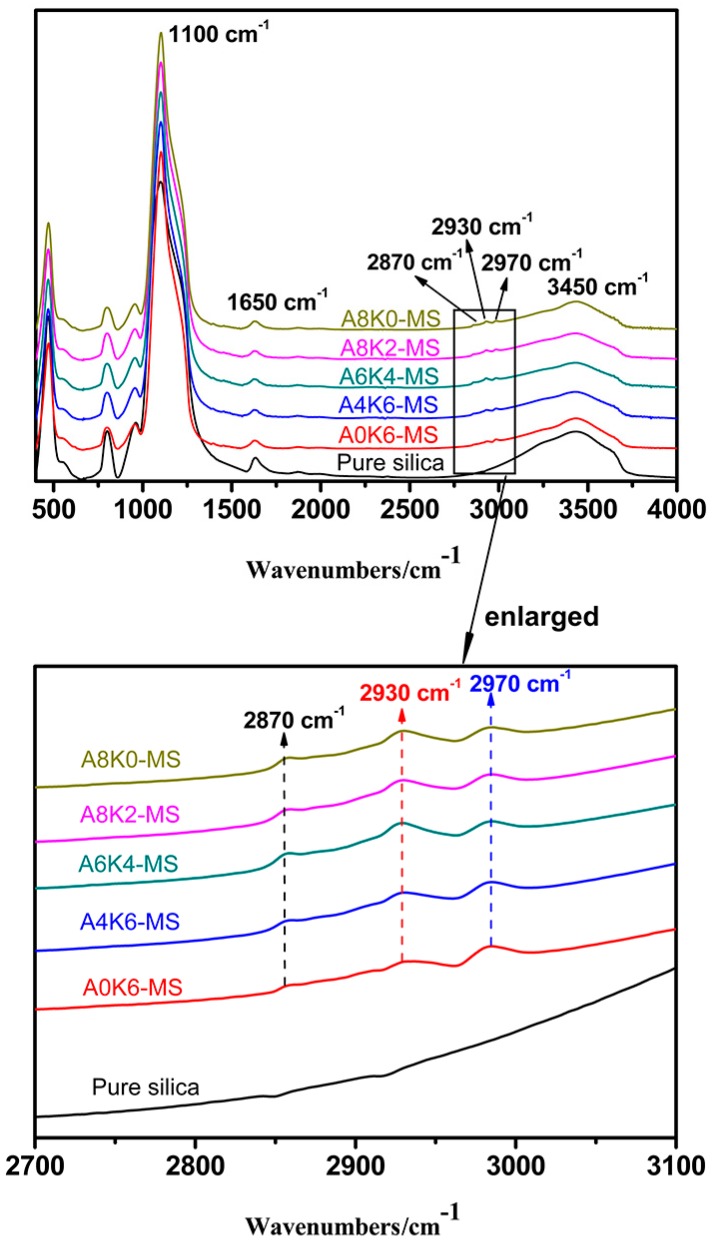
Fourier transform infrared (FT-IR) spectra of pure silica and all modified silica.

As shown in Figure 2, compared to the FT-IR spectra of the pure silica, all curves of the modified silica had absorption peaks at 2930, 2970 and 2870 cm^−1^, attributed to the vibrations of –CH_2_– and –CH_3_ bonds [37]. The appearance of organic groups, such as –CH_2_– and –CH_3_, on the modified silica surface shows that K-MEPTS and AEO exist on the silica surface. 

The absorption peaks at 3450 and 1650 cm^−1^ correspond to the stretching and deforming vibration modes of the –O–H bonds [38], respectively. The relative intensity (RI) of the peak at 3450 cm^−1^, which is the difference between the intensity of the peak and the baseline, is determined by the number of –O–H bonds. Therefore, a high RI means a large number of hydroxyl groups is present on the silica surface. RI can be calculated using the normalized FT-IR data, and the RI of all samples are listed in Table 3. The RI of all silica modified by AEO and K-MEPTS together decreased with increasing the AEO used in silica modification, indicating that replacing K-MEPTS with AEO can more effectively reduce the amount of active –O–H bonds on the silica surface.

#### 3.1.2. Raman Spectroscopy of Pure and Modified Silica

**Figure 3:**
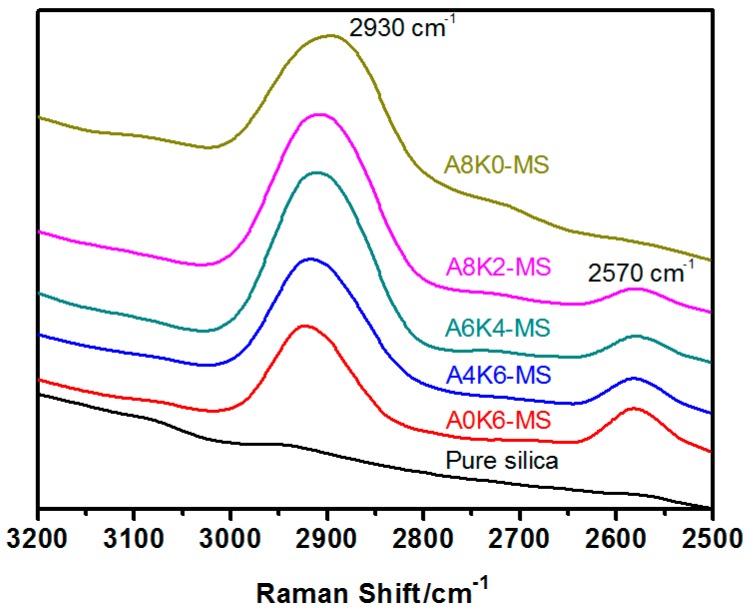
Raman spectra of pure silica and all modified silica.

No peak is shown on the curve of pure silica; therefore, no organic groups are present on the surface of pure silica (Figure 3). Meanwhile, one peak is found on the curve of A8KO-MS and two peaks on other curves. The peak at 2930 cm^−1^ corresponds to methylene bonds, demonstrating that the AEO and K-MEPTS were grafted on the silica surface. Another peak is recorded at 2570 cm^−1^, which corresponds to –S–H bonds, indicating that K-MEPTS was grafted on the silica surface.

The peak intensity around 2570 cm^−1^ for A4K6-MS is significantly weaker than that of A0K6-MS. The only difference for A0K6-MS and A4K6-MS was whether AEO was used in silica modification. The most probable reason for this result was that the AEO molecule had a chance to shield the mercaptopropyl group on K-MEPTS.

#### 3.1.3. TGA Curves of Pure and Modified Silica

**Figure 4:**
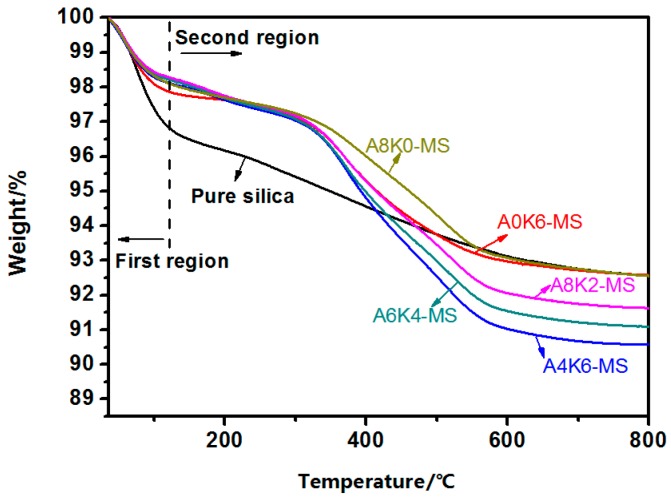
Thermal gravimetric analyzer (TGA) curves of pure silica and all modified silica.

As illustrated in Figure 4 and Table 4, all the samples exhibited large weight losses in the first region between 35 and 120°C. The weight loss in this region was caused by the removal of the adsorbed water. The amount of adsorbed water on the silica surface shows the same tendency as the amount of hydroxyl groups on the silica surface.

All of modified silica had a large weight loss in the second region between 120 and 800 °C. The weight loss of modified silica was due to the degradation of AEO and K-MEPTS, and the weight loss of pure silica was due to the dehydroxylation of hydroxyl groups. All modified silica had a larger weight loss than pure silica in the second region; whereas A4K6-MS had a larger weight loss than A0K6-MS in the second region. This is a noteworthy finding that indicates that both K-MEPTS and AEO were grafted onto the silica surface.

For A4K6-MS, A6K4-MS and A8K2-MS, the total weight of AEO and K-MEPTS grafted on silica decreased, but the amount of adsorbed water decreased sequentially. Therefore, AEO is a more effective modifier than K-MEPTS in changing the hydrophilicity of silica. This result could be attributed to the structure of the AEO molecule, which likely covered multiple hydroxyl groups on the silica surface. In contrast, a K-MEPTS molecule could react with up to one hydroxyl group on the silica surface. The decrease in the hydrophilic nature of silica generally means that the compatibility between silica and the organic phase, such as rubber, is improved, which is crucial for preparing high performance silica/NR composites.

**Figure 5:**
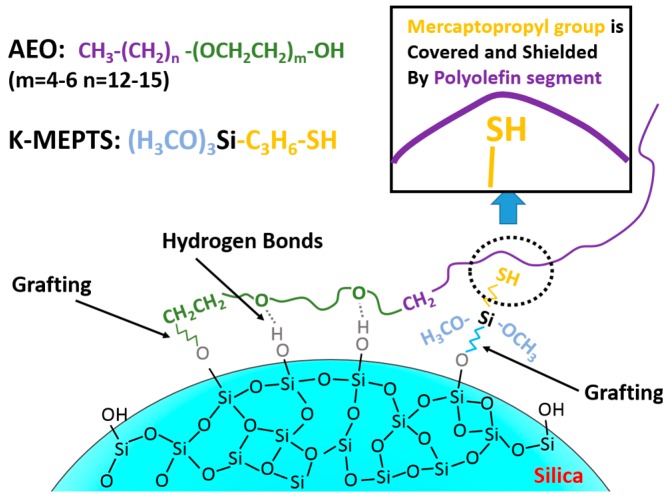
Schematic diagram of the interaction between AEO and K-MEPTS during silica modification.

Based on the above results, the interaction between AEO and K-MEPTS is shown in Figure 5. The activity of the mercaptopropyl on K-MEPTS is affected by this interaction. Therefore, the chargeability and reactivity of silica modified with AEO and K-MEPTS together differ from that of silica modified with K-MEPTS alone.

### 3.2. Characterization of Silica/NR Master Batches Prepared with Pure and Modified Silica

#### 3.2.1. Co-Coagulation of Silica/NR Mixture in Preparing the Master Batches

**Figure 6:**
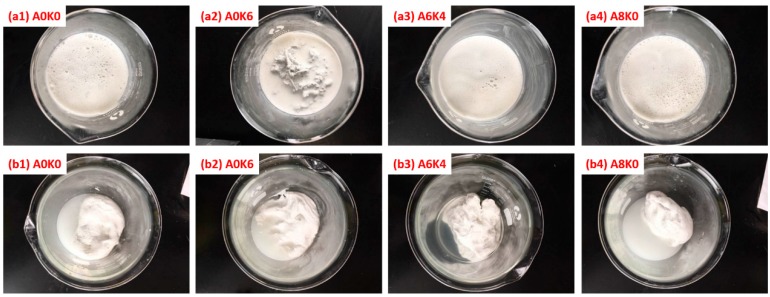
Preparation process of the master batches with pure and modified silica.

The co-coagulation of the silica/NR mixture used in preparing the master batches using several modified silica and pure silica (A0K0-MS) is shown in Figure 6. The images of the silica/NR mixtures, which were prepared by adding the NR latex into the silica slurries and stirring for 10 min, are shown in Figure 6a. Images of master batches, prepared by adding 3% formic acid solution into mixtures, are shown in Figure 6b. A6K4-MS was selected as the representative for silica modified with both AEO and K-MEPTS together, because the phenomenon of preparing silica/rubber master batches was almost the same for A4K6-MS, A6K4-MS and A8K2-MS.

As presented in Figure 6(a2), part of the mixture coagulated when NR was mixed with A0K6-MS, even if the formic acid solution was not added into this mixture. This phenomenon indicates that K-MEPTS promotes the coagulation of NR latex. As presented in Figure 6b, A0K6-MB, A8K0-MB and pure silica masterbatch were largely clustered, and the residual aqueous phase was white and turbid with abundant silica. Conversely, A6K4-MB coagulated as complete sediments, and the residual aqueous phase was clear. 

**Figure 7:**
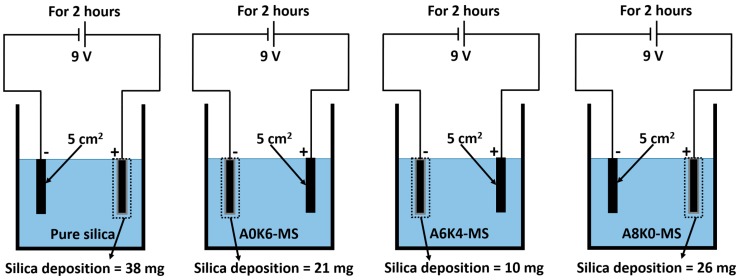
Schematic diagram of the silica deposition experiment.

The difference in the coagulation phenomenon in different master batches was caused by electrostatic attractive or repulsive forces. An additional simple experiment was performed to confirm this inference. As presented in Figure 7, four kinds of pure and modified silica were added to a beaker with a 2 by 2.5-cm copper plate, as an electrode device. After two hours, the silica deposited on the electrode was weighed after drying. The results of silica deposition are also shown in Figure 7. The pure silica and A8K0-MS were deposited on the positive electrode, indicating that both were negatively charged. In contrast, A0K6-MS and A6K4-MS were deposited on the negative electrode; meanwhile, the amount of A0K6-MS deposited was more than the A6K4-MS deposited. This result indicates that A0K6-MS and A6K4-MS were positively charged, and the positive charge of A0K6-MS was more significant than that of A6K4-MS.

**Figure 8:**
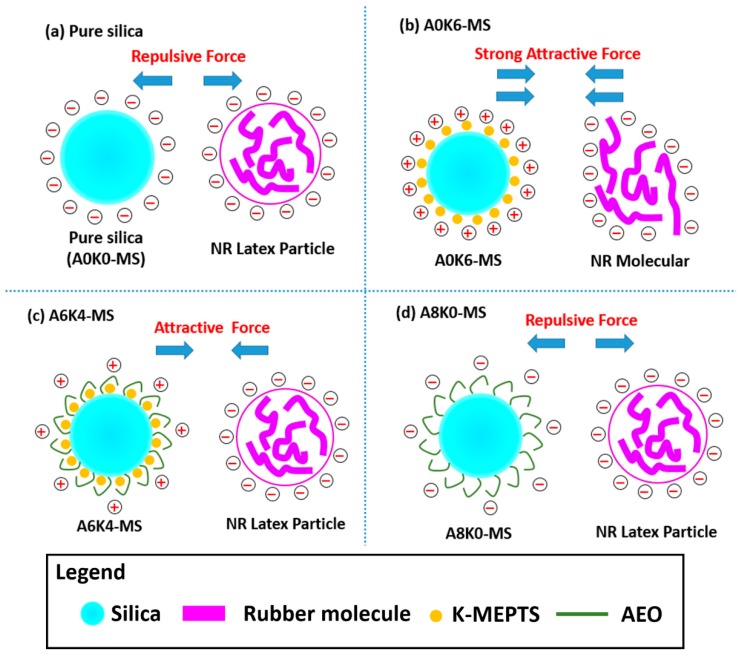
Schematic diagram of the electrostatic forces between pure or modified silica and NR.

Based on the above results, we made a schematic diagram of the electrostatic attractive or repulsive forces between silica and latex particles. The surface of both the silica particle and NR latex particle were negatively charged [39], causing an electrostatic repulsion in the system, as presented in Figure 8a. Therefore, pure silica/NR masterbatch had a large amount of silica loss in the aqueous phase. The changes in the silica surface charge contributed to the adsorption between rubber and silica. However, A0K6-MS had a significantly positive charge, resulting in a strong attractive force between A0K6-MS and rubber latex particles. This force would damage the electrical layer stability of the rubber latex particles, as shown in Figure 8b. Therefore, a mixture of A0K6-MS and NR latex was coagulated in the absence of formic acid solution.

The positive charge of A6K4-MS was weaker than that of A0K6-MS. The attractive force between the NR latex particles and modified silica was at an appropriate level, resulting in the successful preparation of A6K4-MB. However, AEO was a nonionic surfactant that had no effect on the electrical performance of the silica surface. Therefore, a repulsive force still existed between A8K0-MS and NR latex particles, as presented in Figure 8d. In this scenario, the co-coagulation of the mixture of latex, which included NR latex and A8K0-MS, was unsatisfactory.

#### 3.2.2. Actual Amount of Silica in Silica/NR Master Batches

The actual amount of silica in the master batches was calculated using the following equation:

(1) $$\mathrm{Silica}\;\mathrm{content}\;(\mathrm{phr})=\frac{100\times (R_{m}-R_{r})}{R_{s}+S_{A}\times R_{A}+S_{5}\times R_{5}-(1+S_{5}+S_{A})\times R_{m}}$$

where *R_m_*, *R_r_*, *R_s_*, *R_5_* and *R_A_* are the 800 °C weight (%) of master batches, rubber, silica, K-MEPTS and AEO, respectively. *S*_5_ is the weight ratio of K-MEPTS to modified silica, and *S_A_* is the weight ratio of AEO to modified silica. The calculated results according to this equation are shown in Table 5. For all master batches that contained silica modified with K-MEPTS and AEO together, the actual amount of silica in the master batches is approximately equal to the additional amount of silica (50 parts per hundred of rubber (phr)). In contrast, the actual amount of silica in A0K6-MB, A8K0-MB and A0K0-MB is obviously lower than the 50 phr addition of silica. This result further indicates a huge loss of silica occurred during coagulation when K-MEPTS or AEO was used individually to modify silica. The results are consistent with the observed macroscopic phenomena in Section 3.2.1.

Because of the interaction between AEO and K-MEPTS, silica modified with AEO and K-MEPTS together had appropriate chargeability and completely co-coagulated with the rubber, which is another key factor for preparing high performance silica/NR composites using the latex compounding method.

#### 3.2.3. Micromorphology of the Silica/NR Master Batches Observed by TEM

**Figure 9:**
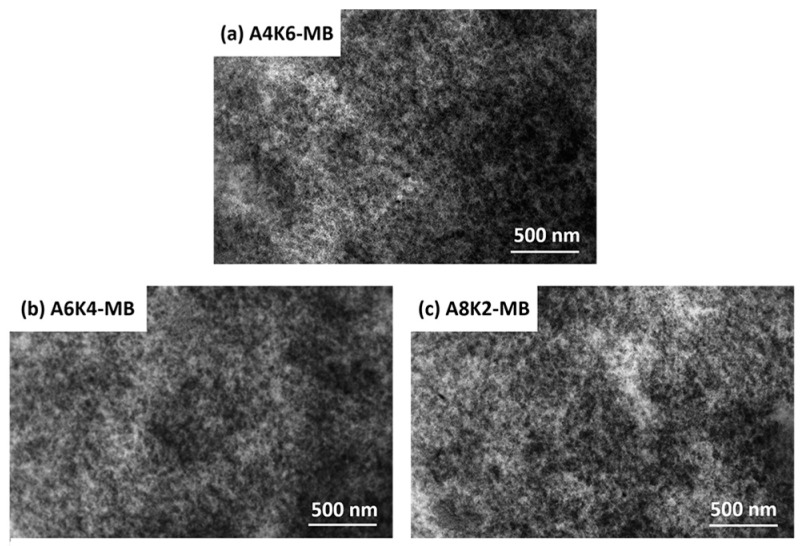
TEM images of (**a**) A4K6-MB; (**b**) A6K4-MB; and (**c**) A8K2-MB.

As shown in Figure 9, silica is uniformly dispersed in the matrix without serious aggregation in these master batches. The silica dispersion in A6K4-MB is somewhat more homogeneous than in other samples. This composite has fewer silica aggregates than the others. This result demonstrates that the electrostatic attractive force between A6K4-MS and rubber molecules is the most successful in the three master batches that were successfully prepared.

### 3.3. Characterization of the Preparation Process of Silica/NR Composites

The method of preparing A4K6-C, A6K4-C and A8K2-C is described in Section 2.4. A0K6-C cannot be prepared using master batches due to the huge silica loss in preparing A0K6-MB. Therefore, the mechanical blending method was used for preparing A0K6-C.

As shown in Table 6, A0K6-C had the shortest scorch time (*T*_10_) in all the tested silica/rubber compounds. In increasing order, the scorch times of A4K6-C, A6K4-C and A8K2-C were longer than that of A0K6-C. The reactivity of the mercaptopropyl group decreases when AEO was used together with K-MEPTS. Therefore, AEO slowed the rate of reaction between K-MEPTS and rubber. The “scorchy: problem can be mitigated by using AEO and K-MEPTS together in silica modification. The Δ*M* of A0K6-C was a little bit higher than that of A4K6-C. The silica modified by K-MEPTS could function as a cross-linking point, resulting in the improved crosslinking density of the silica/rubber composite, which is reflected by Δ*M*. Therefore, AEO has little effect on preventing the reaction between K-MEPTS and rubber.

### 3.4. Characterization of Silica Dispersion in Silica/NR Composites

#### 3.4.1. Payne Effect of Silica/NR Compounds Investigated by RPA

**Figure 10:**
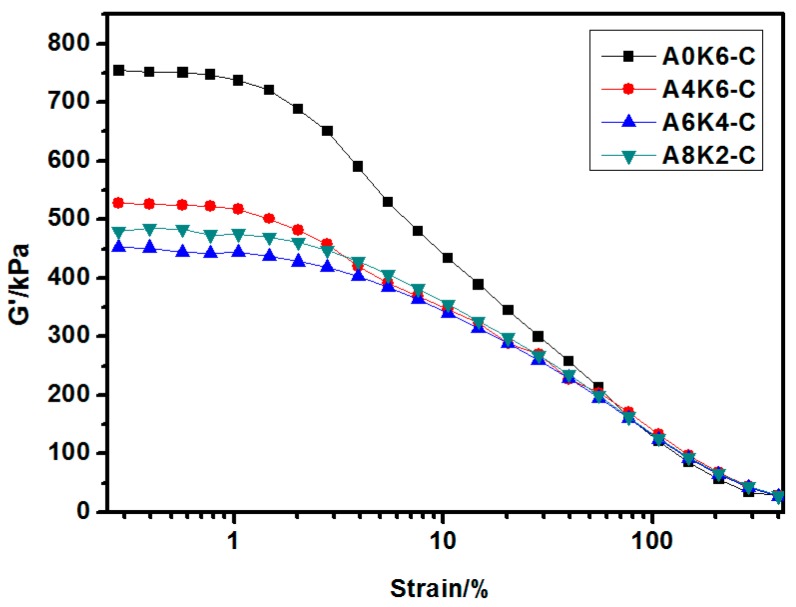
Strain amplitude dependence of the storage modulus (*G*′) of four silica/NR compounds.

At approximately 1% strain, the storage modulus (*G*′) decreases rapidly with increasing strain and approaches 0 kPa with a sufficiently large strain as shown in Figure 10. The Payne effect is indicated by the Δ*G*′, which is the difference between the minimum and maximum *G*′ in the curve [40]. This effect can be attributed to deformation-induced changes in the microstructure of the material. The Payne effect is not significant when Δ*G*′ is small. The low Payne effect indicates high uniformity of the filler dispersion.

As shown in Figure 10, A0K6-C exhibits a more obvious Payne effect than the other silica/NR compounds. This finding indicates that the dispersion of silica modified by AEO and K-MEPTS together in silica/NR composites prepared in master batches is more homogeneous than that of silica modified by K-MEPTS alone in silica/NR composites prepared using mechanical blending. This result was caused by two reasons: AEO effectively reduces the hydrophilicity of silica, resulting in improving silica dispersion in NR matrix; and the silica dispersion in the rubber matrix is improved by the latex compounding method [22].

The Payne effect of the composite decreases with increasing AEO used in the silica modification and then reaches the lowest value when the weight ratio of AEO to silica is 6:100 and that of K-MEPTS to silica is 4:100 (A6K4-C). This result show the same tendency as the silica dispersion in silica/NR master batches. Therefore, the silica dispersion in master batches directly affects the dispersion of silica in silica/NR compounds, which further indicates the importance of preparing silica/NR master batches with a homogeneous silica dispersion.

#### 3.4.2. Micromorphology of the Silica/NR Composites Observed by TEM

**Figure 11:**
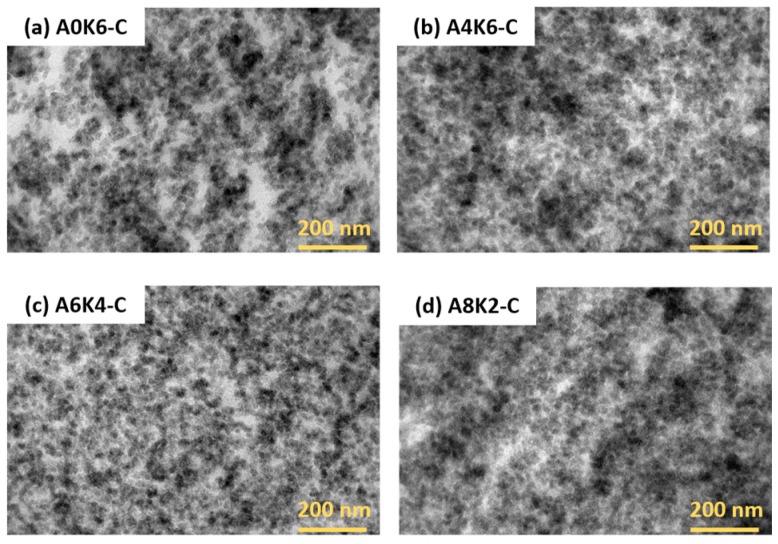
TEM images of four silica/NR composites.

Figure 11 shows that the silica dispersion in the vulcanizates of A4K6-C, A6K4-C and A8K2-C is significantly more homogeneous than in the vulcanizate of A0K6-C, because the latter contains more silica aggregates. AEO grafted onto the silica surface, but did not react with rubber, resulting in the formation of a physical interface between silica and rubber. This physical interface reduced the polarity of silica and improved the compatibility between silica and rubber, thus improving the dispersion of silica in rubber. In contrast, K-MEPTS grafted onto the silica surface and reacted with rubber, resulting in forming a chemical interface between silica and rubber. The silica particles could be connected with the rubber molecules with the help of K-MEPTS. Therefore, this chemical interface played a role in preventing the aggregation of primary silica particles. Silica modified by using both AEO and K-MEPTS together benefits from the chemical and physical interaction between silica and rubber. In this research, the silica/NR composites had the best silica dispersion when the weight ratio of AEO to K-MEPTS was 6:4 for silica modified by 10% weight modifiers.

### 3.5. Characterization of Silica/NR Composites

#### 3.5.1. Mechanical Performances of Silica/NR Composites

**Figure 12:**
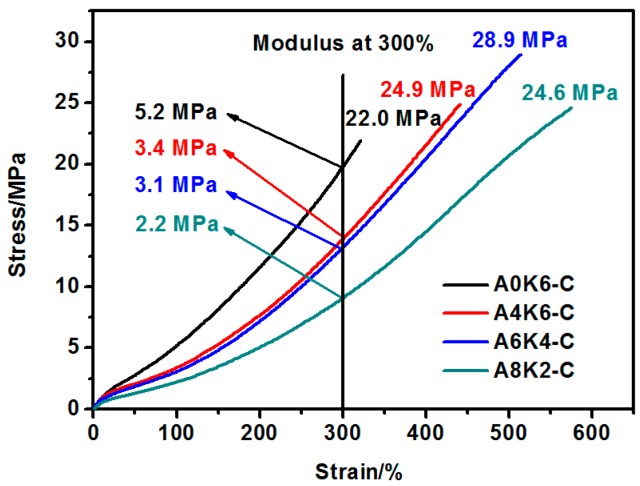
Mechanical performance of silica/NR composites.

As shown in Figure 12 A0K6-C vulcanizate exhibits a 50% higher modulus and a 40% lower elongation at break than A4K6-C vulcanizate. The tensile strength of A0K6-C vulcanizate is 40% lower than that of A4K6-C vulcanizate. The chemical interface formed by K-MEPTS functions as a “coupling bridge” to improve the reinforcing efficiency of silica on rubber, demonstrated by the high modulus. However, the excessive chemical interface between silica and rubber leads to stress-concentrated regions [31], resulting in a low elongation at the break. Because of the physical interface formed by AEO, the most elongated rubber chains slip along the surface of silica and equalize the high stress [41], resulting in a proper modulus and elongation at break of A4K6-C vulcanizate.

For vulcanizates of A4K6-C, A6K4-C and A8K2-C, with decreasing K-MEPTS used in silica modification, the modulus decreases, but the elongation at break increases. The chemical interface between silica and rubber becomes stronger with the increase in the amount of K-MEPTS used in silica modification. The physical interface between silica and rubber becomes stronger with the increase in AEO used in silica modification. As presented in Figure 12, the tensile strength of the composites peaked at a value of 28.9 MPa for the A6K4-C vulcanizate sample. The tensile strength of the silica/NR composite was affected by both the modulus and the elongation at break. Therefore, the mechanical performance of the silica/NR composites were the best when a proper combination of physical and chemical interface existed between silica and NR. In this research, the silica/NR composites had the best mechanical performance when the weight ratio of AEO to K-MEPTS was 6:4 with silica modified by 10% weight modifiers.

#### 3.5.2. Dynamic Performances of Silica/NR Composites

**Figure 13:**
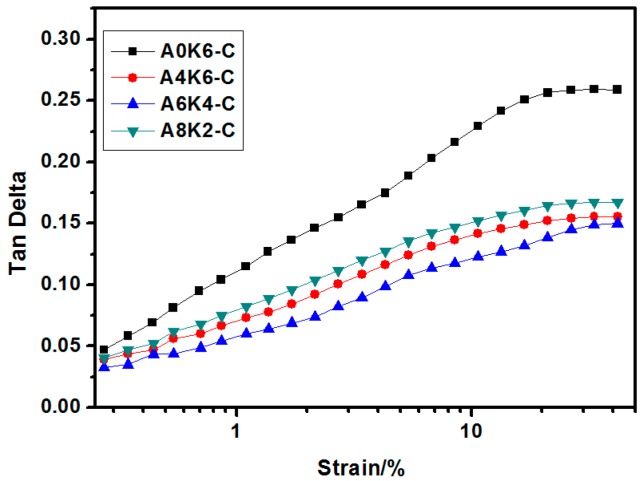
Strain-tan δ curve of four silica/NR composites (60 °C).

In tire applications, the tan δ values at 60 °C are typically used to predict rolling resistance. As presented in Figure 13, the tan δ values under large strain (>5%) are arranged from high to low in the following order: A0K6-C vulcanizate, A8K2-C vulcanizate, A4K6-C vulcanizate and A6K4-C vulcanizate. The rolling resistance of the A0K6-C vulcanizate was the highest of these four silica/NR composites due to the strong mutual friction between silica particles under cyclic reversed loading. In theory, the silica fixed with rubber molecules by a “coupling bridge” would barely enhance internal friction loss [4,27]. However, many silica aggregates were still present in the A0K6-C vulcanizate. The mutual friction remained strong between the silica particles that were tightly conglutinated under cyclic reversed loading. In contrast, silica modified with AEO and K-MEPTS together improved silica dispersion, with silica and rubber fixed together. Therefore, combining good silica dispersion and an appropriate interaction between silica and rubber by designing a proper interface were beneficial to improving the dynamic performance of silica/NR composites.

The relationship of the tan δ of silica/NR composites with increasing strain is also shown in Figure 13. The tan δ value of A0K6-C vulcanizate is the greatest and increases rapidly with increasing strain. The increase of the tan δ amplitude with strain increase in the curves of A4K6-C, A6K4-C and A8K2-C vulcanizates are lower than that of A0K6-C vulcanizate. A6K4-C vulcanizate has the lowest increase in tan δ value with strain in all samples, which means that when using A6K4-C composite as tire tread, the rolling resistance of the tire changes minimally with increasing vehicle load.

**Figure 14:**
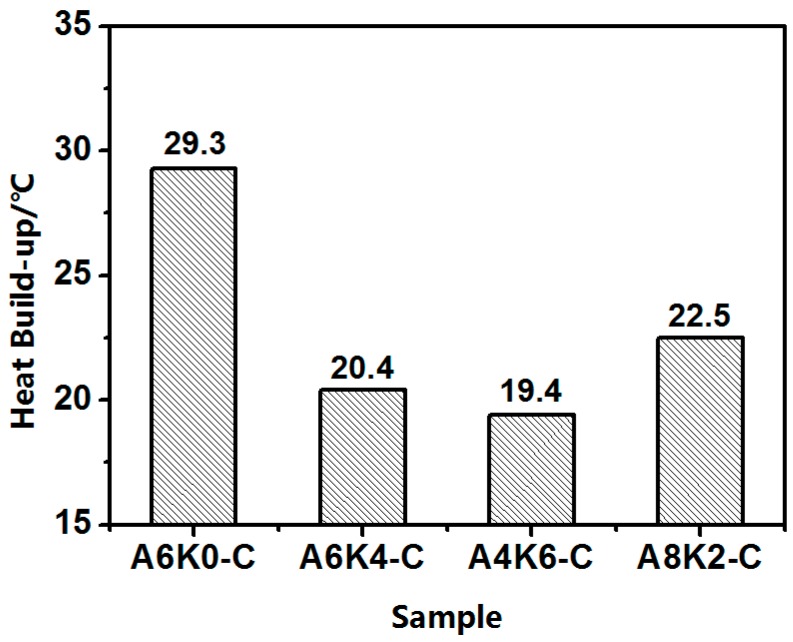
Heat build-up of four silica/NR composites.

As shown in Figure 14, the heat build-up test, used to characterize the rolling resistance of silica/rubber composites in practical applications, was also performed to investigate the dynamic performance of different silica/NR vulcanizates. The heat build-up values are arranged from high to low in the following order: A0K6-C vulcanizate, A8K2-C vulcanizate, A4K6-C vulcanizate and A6K4-C vulcanizate. This result aligns well with the loss factor (tan δ) measured by RPA. Therefore, using AEO and K-MEPTS together for silica modification is a novel strategy for preparing silica/NR composites used in “green tires” with low rolling resistance.

## 4. Conclusions

In this research, we proposed an extremely effective strategy of using both AEO and K-MEPTS together for silica modification, by preparing silica/NR using the latex compounding method. As we designed, tailored silica was dispersed in the aqueous phase and completely co-coagulated with the NR. The silica/NR composites prepared by these master batches had ideal dynamic and mechanical performance, especially for A6K4-C, which was a composite containing silica modified with 6% AEO and 4% K-MEPTS (weight ratio of modifier to silica). This research provides a significant potential for preparing high performance silica/NR composites in a user-friendly method.

Furthermore, we investigated the effect of several interactions on the performance of silica/rubber composites. We confirmed that both AEO and K-MEPTS grafted onto the silica surface, and AEO shielded K-MEPTS, resulting in weakening the chargeability and activity of the mercaptopropyl group on K-MEPTS. This interaction between these two modifiers contributed to solving the problem of huge silica loss and “scorchy” behavior in the preparation of silica/rubber composites. This is a novel and practical interaction confirmed in this work. In silica/NR composites, AEO formed a physical interface between silica and rubber, resulting in reducing the aggregation of silica and improving the silica dispersion in the rubber matrix. Moreover, K-MEPTS formed a chemical interface between silica and rubber, resulting in strengthening the connection between silica and rubber and reducing the mutual friction between silica particles. Finally, AEO and K-MEPTS acted synergistically to improve the mechanical and dynamic performances of silica/NR composites.

Overall, the novel strategy outlined in this article provides a new strategy for the preparation of rubber materials with excellent performance. We hope that the preparation of silica/NR master batches using this strategy will present practical and profound applications in the rubber industry.

## Figures and Tables

**Table 1:** Formulation of modified silica and its label.

Material	A0K6-MS	A4K6-MS	A6K4-MS	A8K2-MS	A8K0-MS
Silica (dry weight)/g	100	100	100	100	100
3-mercaptopropyltriethoxysilane (K-MEPTS)/g	6	6	4	2	0
polyoxyethylene ether (AEO)/g	0	4	6	8	8

**Table 2:** Formulation of silica/natural rubber (NR) compounds.

Material	Amount (phr ^a^)	Comment
Master batches	155	Filler and matrix
Stearic acid	2.0	Activator
Zinc oxide	5.0	Activator
*N*-1,3-dimethylbutyl-*N*′-phenyl-p-phenylenediamine	2.0	Antioxidant
*N*-Cyclohexyl-2-beozothiazole sulfenamide	2.0	Accelerator
1,3-Diphenylguanidine	1.0	Accelerator
Sulfur	2.0	Curing agent

^a^ Parts per hundred of rubber.

**Table 3:** Relative intensity (RI) of the peak at 3400 cm^−1^ for pure silica and all modified silica.

Sample	Pure silica	A0K6-MS	A4K6-MS	A6K4-MS	A8K2-MS	A8K0-MS
RI	0.318	0.211	0.189	0.184	0.178	0.196

**Table 4:** Weight losses of pure and modified silica in the first and second regions.

Sample	Weight loss in the first region (35–120 °C)/%	Weight loss in the second region (120–800 °C)/%
Pure silica	3.16	4.27
A0K6-MS	2.13	5.31
A4K6-MS	1.88	7.54
A6K4-MS	1.78	7.13
A8K2-MS	1.71	6.66
A8K0-MS	1.90	5.55

**Table 5:** Weight losses of six kinds of silica/NR master batches.

Samples	Weight residual/%	Theoretical amount of silica in masterbatches/phr	Actual amount of silica in masterbatches/phr
Pure NR	1.32	-	-
Pure silica	92.57	-	-
KH-590	9.13	-	-
AEO	3.27		
A0K6-MB	26.32	50	38.33
A4K6-MB	30.76	50	49.55
A6K4-MB	30.83	50	49.83
A8K2-MB	30.65	50	49.46
A8K0-MB	23.71	50	33.31
A0K0-MB	22.89	50	30.96

**Table 6:** Vulcanization characteristics of four kinds of silica/NR compounds.

Samples	*T*_10_/min	*T*_90_/min	Δ*M*/dNm
A0K6-C	0.65	3.03	30.39
A4K6-C	2.05	5.28	28.72
A6K4-C	3.25	5.87	26.89
A8K2-C	3.72	7.33	24.95
